# Peru – Progress in health and sciences in 200 years of independence

**DOI:** 10.1016/j.lana.2021.100148

**Published:** 2021-12-20

**Authors:** Rodrigo M. Carrillo-Larco, Wilmer Cristobal Guzman-Vilca, Fabiola Leon-Velarde, Antonio Bernabe-Ortiz, M. Michelle Jimenez, Mary E. Penny, Camila Gianella, Mariana Leguía, Pablo Tsukayama, Stella M. Hartinger, Andres G. Lescano, María Sofía Cuba-Fuentes, Yuri Cutipé, Francisco Diez-Canseco, Walter Mendoza, Cesar Ugarte-Gil, Andrea Valdivia-Gago, Carol Zavaleta-Cortijo, J. Jaime Miranda

**Affiliations:** a Department of Epidemiology and Biostatistics, School of Public Health, Imperial College London, St Mary's Campus, London, United Kingdom; b CRONICAS Center of Excellence in Chronic Diseases, Universidad Peruana Cayetano Heredia, Lima, Peru; c Departamento de Ciencias Biológicas y Fisiológicas, Facultad de Ciencias y Filosofia, Universidad Peruana Cayetano Heredia, Lima, Peru; d United Nations Children's Emergency Fund (UNICEF), Lima, Peru; e Instituto de Investigación Nutricional, Lima, Peru; f Departmento de Psicología, Facultad de Psicología, Pontificia Universidad Católica del Perú, Lima, Peru; g Chr. Michelsen Institute, Bergen, Norway; h Laboratorio de Genómica, Pontificia Universidad Católica del Perú, Lima, Peru; i Laboratorio de Genómica Microbiana, Facultad de Ciencias y Filosofía, Universidad Peruana Cayetano Heredia, Lima, Peru; j Parasites and Microbes Programme, Wellcome Sanger Institute, Hinxton, United Kingdom; k Clima, Latin American Center of Excellence for Climate Change and Health, Universidad Peruana Cayetano Heredia, Lima, Peru; l University of Basel, Basel, Switzerland; m Department of Epidemiology and Public Health, Swiss Tropical and Public Health Institute, Basel, Switzerland; n Emerge, Emerging Diseases and Climate Change Research Unit, School of Public Health and Administration, Universidad Peruana Cayetano Heredia, Lima, Peru; o Department of Medicine, School of Medicine, Universidad Peruana Cayetano Heredia, Lima, Peru; p Ministry of Health, Lima, Peru; q Fondo de Población de las Naciones Unidas (UNFPA), Lima, Peru; r Instituto de Medicina Tropical “Alexander von Humboldt”, Universidad Peruana Cayetano Heredia, Lima, Peru; s TB Center, London School of Hygiene and Tropical Medicine, London, United Kingdom; t Center for Global Health, Perelman School of Medicine, University of Pennsylvania, Philadelphia, United States; u Faculty of Public Health and Administration, Universidad Peruana Cayetano Heredia, Lima, Peru; v Intercultural Citizenship and Indigenous Health Unit (UCISI), Universidad Peruana Cayetano Heredia, Lima, Peru; w Nutritional Epidemiology Group, School of Food Science and Nutrition, University of Leeds, Leeds, United Kingdom; x The George Institute for Global Health, University of New South Wales, Sydney, Australia; y Department of Non-Communicable Disease Epidemiology, Faculty of Epidemiology and Population Health, London School of Hygiene and Tropical Medicine, London, United Kingdom

**Keywords:** BMI, body mass index

## Abstract

Peru celebrates 200 years of independence in 2021. Over this period of independent life, and despite the turbulent socio-political scenarios, from internal armed conflict to economic crisis to political instability over the last 40 years, Peru has experienced major changes on its epidemiological and population health profile. Major advancements in maternal and child health as well as in communicable diseases have been achieved in recent decades, and today Peru faces an increasing burden of non-communicable diseases including mental health conditions. In terms of the configuration of the public health system, Peru has also strived to secure country-wide optimal health care, struggling in particular to improve primary health care and intercultural services. The science and technology infrastructure has also evolved, although the need for substantial investments remains if advancing science is to be a national priority. Climate change will also bring significant challenges to population health given Peru's geographical and microclimates diversity. Looking back over the 200-years of independence, we present a summary of key advances in selected health-related fields, thus serving as the basis for reflections on pending agendas and future challenges, in order to look forward to ensuring the future health and wellbeing of the Peruvian population.

**Resumen (translated abstract):**

El Perú cumple 200 años de independencia en 2021. Durante estos dos siglos de vida independiente, junto con periodos sociales y políticos turbulentos, incluyendo un conflicto armado interno, hiperinflación y la inestabilidad política de los últimos 40 años, el Perú ha experimentado importantes cambios en su perfil epidemiológico con repercusiones directas en la salud de la población. En las últimas décadas, los indicadores de salud materno-infantil y de las enfermedades transmisibles muestran mejoría importante, pero el país se enfrenta de manera simultánea a una carga cada vez mayor de enfermedades no transmisibles y de salud mental. En cuanto a los sistemas de salud pública, se han realizado esfuerzos por aumentar la cobertura y calidad de la atención de salud en todo el país, apostándose en particular por mejorar la atención primaria. La ciencia y tecnología relacionadas con la salud también han mejorado, aunque si se quiere que la ciencia sea una prioridad nacional, son necesarias inversiones sustanciales. El cambio climático traerá importantes desafíos para la salud de la población, dada la diversidad geográfica y de microclimas del país. Para conmemorar los 200 años de vida independiente del Perú, presentamos un resumen de avances clave en diversas áreas y temas relacionados con la salud. Este repaso sirve como base para reflexionar sobre agendas y desafíos pendientes y futuros, con el fin de asegurar la salud y el bienestar de la población peruana en las próximas décadas.

## Introduction

In 2021 Peru celebrates 200 years of independence. Peru is home to 33 million people, and a large share of its population is concentrated in its capital city, Lima, with 10 million people; the remaining population lives in coastal, Andean and Amazonian rainforest areas. Indigenous populations represent seven million, and 55 languages are spoken in the country.^1^^,^^2^

**Figure 1:**
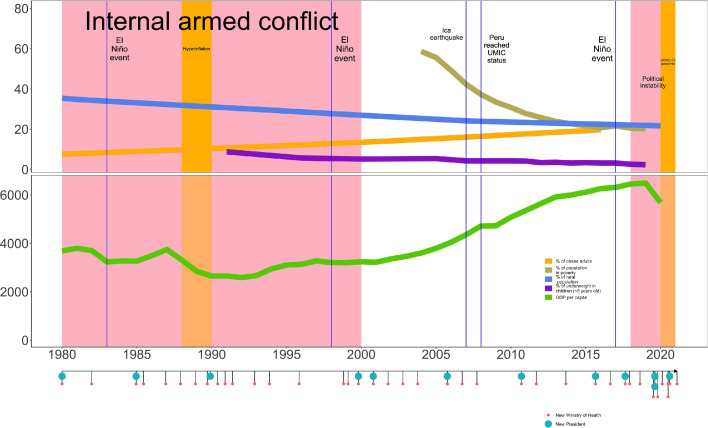
Historical timeline of social, environmental, and political events impacting health in Peru showing number of presidents and health ministers alongside health and economic metrics.

Peru has experienced multiple environmental, socioeconomic and political challenges that have directly impacted population's health. Peru has had several political crises with nine *coup d'etats* between 1900 and 2021,^3^ and has seen 5 presidents and 11 Ministers of Health between July 2016 and July 2021.^4^ The 20-year internal armed conflict spanned from 1980 to 2000 had a toll of ∼70,000 deaths,^5^ and continuous socioeconomic crises hit the country including repeated hyperinflation in the 1980s and 1990s. There have been social conflicts like the *Baguazo*, in 2009, when 34 people including ten Indigenous civilians died.^6^ These convoluted series of events (Figure 1) in the unique social and political Peruvian context are fertile soil for syndemics to thrive.^7^

Despite the challenges faced in its recent history, research, science and innovation have also made important progress in the country,^8^ including key successful exemplars that have informed health and healthcare delivery in Peru and globally. One of these, an example among many, is the microscopic-observation drug-susceptibility (MODS),^9^ a tool for accurate and faster tuberculosis diagnosis.

In this review, rather than delving in detail into particular topics, we provide a brief summary of Peru's health gains and scientific advancements across a range of selected topics. This work was planned as a comprehensive overview of multiple and diverse fields related to epidemiology, public health and science in Peru. This review is a primer where local, regional, and global researchers, practitioners and policymakers, can gain an understanding about past, present and future of Peru's health and science profile.

## Epidemiological transition

### Historic view

Based on national averages Peru has gone through a remarkable progress in some of the major indicators of population health during the last century. The 1940 census counted ∼8 million inhabitants. Eight decades later, its population increased by fourfold, infant mortality dropped from 160 per 1,000 live births in 1950 to 12.6 per 1,000 live births in 2019.^10^ Life expectancy at birth rose from 43.8 to 76.8 years during the same period; and at age 60, remaining life expectancy was 13.3 and 14.3 years for males and females by the early 50s, and before the COVID-19 pandemic it reached 20.7 and 23.8 years, respectively.^10^

### Drivers of these changes

While most of these achievements have been explained by development interventions, e.g., education, sanitation, urbanization, and expanded health services and vaccination, it has also been integrated into a narrative of continued health progress. Most transition analyses in Peru have been based on mortality trends, as caused by single specific entities. What is still missing from this approach is the incorporation of the role of morbidity and disabling conditions, and most importantly, the role of co-morbidities. What might be more relevant in Peru's epidemiological transition is the role of comorbidities, including communicable and non-communicable diseases coexisting in the same individuals, e.g., diabetes and tuberculosis.^11^

### Looking beyond the COVID-19 pandemic

Peru still lagged behind an advanced stage in the epidemiological transition, and more so if we consider that sub-national transitions hide inequalities. The seemingly simplistic narrative of “health progress”, when progress is expressed as change in profiles through the lens of the epidemiological transition, should not be based only on analysis of mortality records.^12^ A country currently experiencing various simultaneous transitions, with multiple ethnicities and increased social inequalities, cannot be approached as a unique entity without considering multi-morbidity. Understanding the health determinants, beyond those exclusively related with health sector, could explain, among other health issues, why COVID-19 hit so harshly the country. It is essential to overcome the pandemic long-term effects regarding both the long-COVID-19 clinical features and broader societal disruptions, including those caused to the entire health system.

## The Peruvian health system

### The healthcare system in Peru

**Figure 2:**
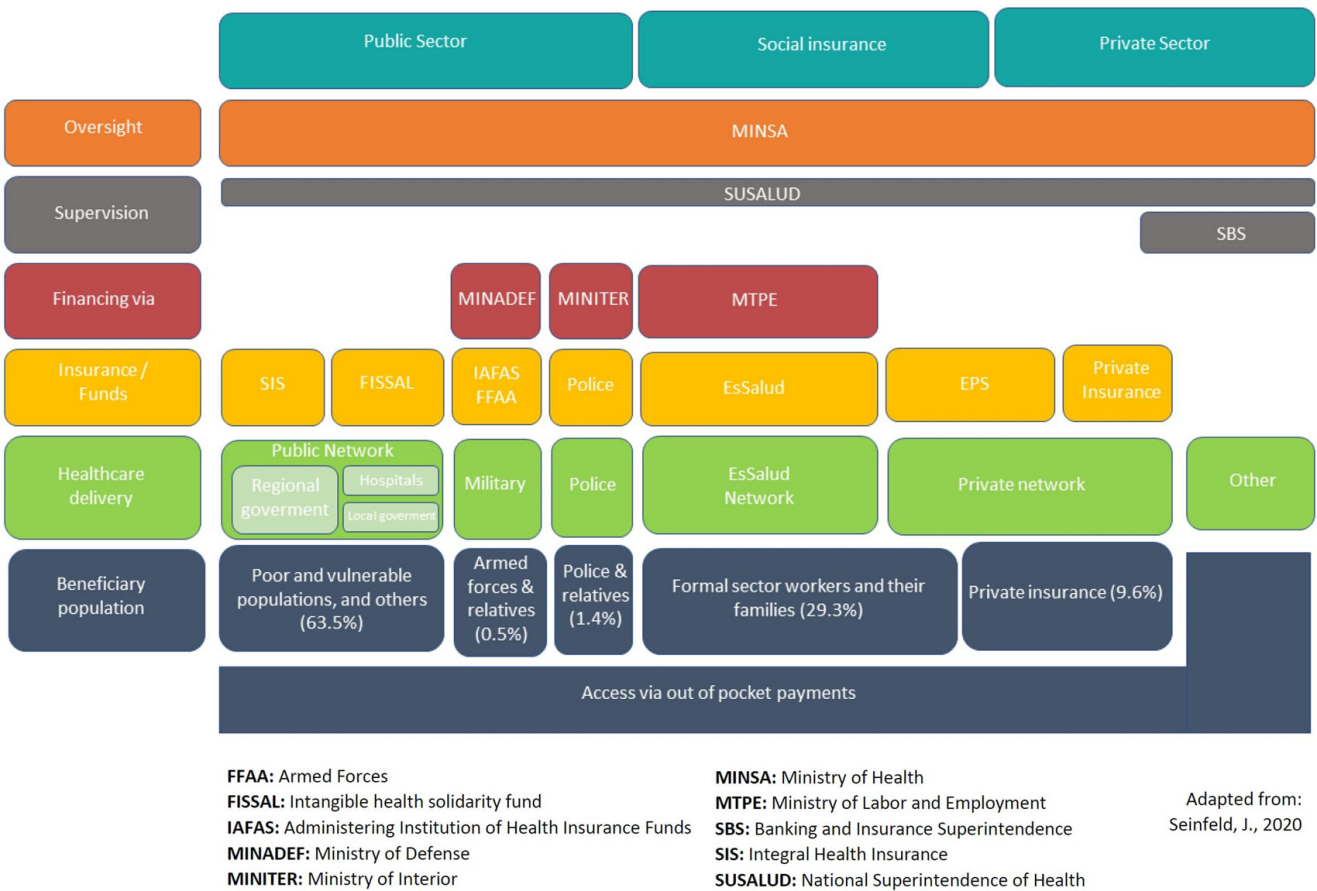
Structure of the Peruvian health system.^13^

The Peruvian health system remains fragmented and segmented in terms of its organization and structure, thus severely constraining the state's capacity to deliver high quality health care for all. The fragmentation is expressed on different funding sources, a diversity of insurance schemes with different coverages, and multiple health service delivery channels (Figure 2).^13^

The financing of the health system is complex and service provision is linked to different insurance schemes, with four providers being the most salient ones. First, the *Seguro Integral de Salud* (SIS) is funded through general taxes, and mostly targets those in poverty to provide free healthcare for certain conditions. As of 2020, ∼65% of the Peruvian population was covered by SIS.^14^ Second, EsSalud, is a contributive service that provides health care, as well as pension and welfare coverage, financed through payroll discounts and is dependent of the Ministry of Labour. Third, the Army and Police Forces are financed through budget derived from the Ministry of Defence. Fourth, there are several types of private insurance that covers a small fraction of the population, ∼10%, with some overlap with EsSalud.^14^

### Lack of unification across providers

Health insurance schemes are linked to different coverages (healthcare basket). Although by law all Peruvians must have access to *the Plan Esencial de Salud* (PEAS – Basic Health Plan) as part of the SIS insurance, EsSalud covers more health conditions and procedures than the SIS.^15^^,^^16^ This fragmentation is also reflected on the provision of health care and organization of the health services. Accessibility to the health service depends heavily on the insurance scheme and –to a lesser extent– geographic location or availability of the services, making the fragmented healthcare system accountable for furthering inequalities.

### Pending tasks

Accessibility and availability of health services differs between rural and urban areas, with hospital services overwhelmingly concentrated in urban areas, and also between regions, making it impossible, especially for people in rural areas, to timely access health care despite their insurance status.^17^^,^^18^ Strengthening the primary health system, along with strong primary prevention programs, could reduce the burden on hospitals capacity.

Little has been done to unify the funding sources and insurers. COVID-19 pandemic has unveiled major problems caused by this fragmentation. One example was the delay to transfer patients from public hospitals (SIS) to EsSalud facilities, where they had better resources including intensive care. Also, the detrimental impact of this segmentation was evident at the beginning of the COVID-19 vaccination campaign:^19^ in March 2021 EsSalud started to vaccinate its population aged ≥80 years with vaccines supplies purchased by the Peruvian Government,^20^ while those covered by SIS in the same age bracket had to wait more days.^21^

## Primary health care

### Current situation

Half of the primary care centers do not have a doctor,^22^ nine out of ten have inadequate infrastructure, and eight out of ten do not have Internet.^23^ Furthermore, about 32% of people eligible for coverage under the SIS insurance attend a healthcare center and out-of-pocket health expenses remains high.^24^ Of note, postgraduate medical training in Family and Community Medicine is not mandatory for primary care practice in Peru.

### Gaps in primary health care

Having a fragmented health system does not allow for the necessary attributes of primary health care to be expressed, namely access, comprehensiveness, and coordination.^25^ This adversely affects the functioning of the health system and achievement of universal health coverage.

There are 8,900 public primary care centers in the country.^22^ Despite this network of primary health centers, in 2019, 41% of Peruvians first accessed a pharmacy when they perceived a health need, and only 29% accessed a primary health center first.^23^

The current epidemiological profile in Peru shows a higher prevalence of non-communicable diseases than communicable diseases. However, primary health centers have insufficient capacity to provide comprehensive care for non-communicable diseases and other chronic infectious diseases, like tuberculosis and HIV. This, because chronic diseases often require linkage and coordination with specialized care, which requires an integrated referral system. Most primary health centers in Peru still use paper records, and the coordination between primary care and the rest of the health system remains inefficient or non-existent.^26^

### Future steps

Changes are required to achieve the goal of universal health coverage with strong primary care as a foundational attribute of a high-quality health system. To achieve this goal, we propose four recommendations. First, to increase the resolution capacity so primary health centers become responsive to the current epidemiological profile and build healthcare networks to ensure efficient delivery of services. Second, to encourage and support the training and retention of family doctors and multidisciplinary teams in primary care facilities. Third, favour the optimal utilization of technologies, including digital technologies, to ensure that all facilities access the Internet and have electronic medical records. Fourth, to achieve adequate financing mechanisms that guarantees continuous and integral care of all Peruvians, regardless of their insurance coverage, socioeconomic status or ethnicity.

## Indigenous populations

### The intercultural challenge for the Peruvian health system

Peru hosts seven million Indigenous people from 55 different ethnicities, 51 from the Amazon and four from the Andes.^1^^,^^2^ Over the past 20 years, two main changes observed in census records. First, the number of Amazon Indigenous communities has increased by ∼50%, from 1,786 communities reported in the 2007 national census, to 2,703 communities in 2017.^27^ Second, Indigenous people have increased their presence in urban locations, while decreasing in rural areas (Table 1).^27^, ^28^, ^29^, ^30^ This is consistent with what has been observed at a national level: people from rural locations have migrated to urban and most prosperous regions.^31^ It follows that the Peruvian health system should incorporate intercultural and cultural safe strategies. Peruvian Indigenous population have non-Western concepts of health, disease, and illnesses, and they also practice their own curative process including the wellbeing of their communities, a close participation of family members in health decisions, consumption of medicinal plants, special food preparations and diet restrictions.^32^, ^33^, ^34^ The treatment is also typically led by an specialist healer (called in Spanish “*curanderas y curanderos*”, “*parteras y parteros*”, “*shaman*”, “*sabio y sabia*”, among others).^35^^,^^36^ All these elements are not yet implemented in the health system; in fact, Indigenous people reported discrimination and exclusion of their practices^37^^,^^38^ which prevent them from receiving an appropriate health care service.

**Table 1:** Change in population size of Indigenous populations.

Reference 2007	Reference 2017	Indigenous population	Year 2007	Year 2017	Change in Population numbers
-	a^162^	Amazon Indigenous communities	1,786	2,703	917
d^30^	b^28^	Urban Quechua	1,521,391	1,578,657	57,266
d^30^	b^28^	Urban Aymara	189,525	198,927	9,402
d^30^	c^29^	Urban Amazon	39,337	56,337	17,000
d^30^	b^28^	Rural Quechua	1,740,359	1,315,013	-425,346
d^30^	b^28^	Rural Aymara	244,845	193,301	-51,544
d^30^	c^29^	Rural Amazon	183,857	156,486	-27,371

Reference 2007 based on mother language for people aged 5+ years; reference 2017 based on self-identification for people ages 12+ years.

(a) Instituto Nacional de Estadística e Informática. Resultados Definitivos del III Censo de comunidades Nativas 2017 [Internet]. Vol. 1. Lima: Gobierno del Perú; 2018 [cited 2021 Jul 10]. Available from: https://www.inei.gob.pe/media/MenuRecursivo/publicaciones_digitales/Est/Lib1598/;

(b) Instituto Nacional de Estadística e Informática. Población Indigena y Originaria de los Andes. In: La autoidentificación Étnica: Población Indígena Censos Nacionales 2017 [Internet]. Lima: Gobierno del Perú; 2018. Available from: https://www.inei.gob.pe/media/MenuRecursivo/publicaciones_digitales/Est/Lib1642/cap03_01.pdf

(c) Instituto Nacional de Estadística e Informática. Población Indígena y Originaria de la Amazonía. In: La autoidentificación Étnica: Población Indígena Censos Nacionales 2017 [Internet]. Lima: Gobierno del Perú; 2018. Available from: https://www.inei.gob.pe/media/MenuRecursivo/publicaciones_digitales/Est/Lib1642/cap03_02.pdf

(d) Instituto Nacional de Estadística e Informática, PNUD - Programa De Las Naciones Unidas Para El Desarrollo, Fondo de Población de las Naciones Unidas. Censo Nacionales 2007: XI de Población y VI de Vivienda: Perfil Sociodemográfico del Perú. [Internet]. Lima: Gobierno del Perú; 2008. Available from: https://www.inei.gob.pe/media/MenuRecursivo/publicaciones_digitales/Est/Lib1136/libro.pdf

Indigenous people also experience major disadvantages such as lack of access to high quality health services, and lack of information disaggregated with an ethnic variable to allow metrics and indicators to monitor the health and wellbeing, including changes over time, of Indigenous populations. In Peru, a Sectoral Policy on Intercultural Health was approved in 2016, aimed to “regulate the actions of intercultural health at the national level, in order to achieve health care as a human right, which promotes inclusion, equity and equal opportunities for male and female Peruvian citizen”.^39^ However, a full implementation of this policy has not yet been accomplished, and therefore, effective results are still far from being achieved.

Only one-third of Indigenous communities in the Amazon have access to a health post,^40^ and in Puno, located in the south Andean region, most of the population did not have access to screening for non-communicable diseases.^41^ Furthermore, the COVID-19 pandemic has made evident the exclusion and abandonment of Indigenous health in Peru expressed by the lack of preparedness to provide adequate and opportune diagnostic test, health care facilities and specialized treatment to severe cases. The availability of data to understand the effects of the pandemic on Indigenous people in the Amazon and the Andeans have been virtually absent especially during the first year of the pandemic, and some reports indicate that Indigenous people in the Amazon experienced a higher level of mortality compared with the general population.^42^ Data generated from Indigenous organizations showed the fatality rate was three times higher for Indigenous people compared with the general population in the Ucayali region.^43^ This information disaggregated by ethnicity is only available in very few areas, and officially there is no consensus about the effect of the pandemic on the health and wellbeing of Indigenous people.^44^

### Socio-environmental determinants of Indigenous health

Health conditions affecting Peruvian Indigenous people and their determinants are not fully understood, and most of the information has been reported by some studies indicating that both, infectious and chronic conditions are present among adult Indigenous people.^45^^,^^46^ Despite the nationwide progress observed on improving chronic undernutrition (see section on Population Nutrition), nutritional deficiencies have been reported to disproportionally affect Indigenous than non-Indigenous children in the Amazonas region.^47^ Reasons that explain the nutritional disadvantages among Indigenous populations are complex, and the compounded effects of lack of potable water, poverty, exposure to heavy metals, and imposed changes to Indigenous diet are among the potential explanatory factors.^48^, ^49^, ^50^ Amazon Indigenous diet was generally described as based on natural local resources including crops (e.g., cassava, maize, and plantain), fish, game, domestic animals, seasonal fruits and insect.^51^, ^52^, ^53^, ^54^ However, most of the food interventions develop to benefit the food security among these populations have not being differentiated of the rest of the general population.^55^

Peru reaches the bicentenary with information that indicates that Indigenous people depend deeply on natural ecosystems for their food, nutrition and health systems.^36^^,^^55^^,^^56^ The health programs and other initiatives and interventions such as food and feeding programs, conducted in Indigenous lands must consider the Indigenous knowledge, practices and technology to foster a genuine complementary and truly intercultural health system.^57^

Moreover, given that Peru is one of the countries in South America with recently contacted Indigenous people or Indigenous who voluntarily self-isolated,^58^ the right of these populations to not be contacted needs to be acknowledged by the health system, and by multiple government actors that work and/or have responsibilities to protect the health of these remote areas. In the past, initial encounters between populations from different locations and cultures have triggered major health outbreaks in the Peruvian Amazon. For example, accelerated economic development has been associated with outbreaks of smallpox and measles, as well as with environmental pollution and death among Indigenous people.^59^, ^60^, ^61^, ^62^ The presence of these populations in the Peruvian Amazon implies that, any interference in the Amazonian land, such as new roads, the extraction of natural resources and construction of dams, without clear regulations and contingency plans, presents major threats most likely to affect the health of the Indigenous populations.

## Population nutrition

### From undernutrition to overnutrition

Peru is recognized for its success in reducing chronic malnutrition in children under five years of age, from a national rate of 23% in 2010 to 12% in 2020.^63^ This reduction was higher in children from the lowest wealth quintile, from rural areas and those with the least educated mothers; and was associated with improved maternal and new-born health care, increased maternal body mass index (BMI), increased average number of years in maternal education, reduced fertility, and migration to urban areas.^64^ These changes have been attributed to sustained political will to implement evidence-based interventions coupled with a period of continued economic growth.^64^ Despite this success, the country still faces nutritional challenges: 40% of children aged 6–35 months are anaemic,^63^ and school-aged children and adolescents have high rates (∼64%) of overweight and obesity.^65^^,^^66^ Data from 2017 to 18 showed that 22% of children aged 6–13 years were overweight and 16% were obese, and for adolescents aged 12–17 years the rates were 20% and 6%, respectively.^66^

### Nutritional transition and drivers

Peru has experienced a nutritional transition for at least three decades. A recent analysis showed stunting decreased while obesity increased among all social groups between 1992 and 2017. The decrease in stunting has been more pronounced in urban compared to rural settings (-4% vs. -2%), while obesity increased more rapidly in rural compared to urban settings (8% vs. 2%).^67^ In addition, obesity prevalence among extremely poor women living in rural areas increased at a faster rate than that of other groups.^67^ These shifts have influenced the profile of the double burden of malnutrition where the basic pattern has shifted from one of undernourished children whose mothers have a ‘normal’ BMI, to one where now most children have a ‘normal’ or healthy anthropometric status, but whose mothers are overweight or obese.^68^ The problem is compounded with patterns of geographical disparities, as most of the cases of the double burden of malnutrition remain concentrated in least urbanized settings.^69^

These changes are supported by studies showing dietary diversity among Peruvian adolescents increased between 2006 and 2013 and disparities in dietary diversity associated with household wealth and place of residence declined. The increase in dietary diversity was driven by higher consumption of animal source foods at the expense of plant based protein such as legumes and pulses, while added sugar consumption remained stable over time, with most adolescents rural and urban reporting its consumption.^70^ In general, evidence has shown an increase in energy consumption from high calorie dense foods along with a decrease in energy consumption from healthy foods in most social groups.^70^

### Nutrition policies

To address these continuous challenges, Peru's Law on “The Promotion of Healthy Foods for Children and Adolescents” (Law 30021) was approved in 2013.^71^ However, implementation rules for the law were not approved until 2017. This law aims to protect and promote the right to public health, with a focus on children and adolescents to reduce diseases related to overweight, obesity and chronic non-communicable diseases. It has a broad scope that includes actions in schools and the community, with the private sector and an “Observatory for Nutrition and the Study of Overweight and Obesity” responsible for coordinating and monitoring implementation. Some progress has been achieved, for example, guidelines for school kiosks, canteens and cafeterias were approved in 2019 and 2020,^72^ new Food Guides for the Peruvian Population were launched in 2019,^73^ and front-of-package labeling for processed foods was approved and implemented in 2019.^74^ The approvals of the implementation rules and their subsequent application have faced political challenges, particularly the front-of-package labeling. Although these policies and guidelines represent important advances, the implementation of these and other necessary policies with a systematic approach that incorporates not only the health, education, and social protection sectors but also the private sector, still requires significant support to translate into changes in dietary behavior and nutrition at a population level.

## Maternal and reproductive health

### Trends and current situation

In last decades Peru has continued to expand its maternal care services, reaching ∼94% of births attended by skilled professionals at a health facility. In rural populations, by the year 2000 <20% of births were attended by a qualified professional, and by the year 2020 it was ∼84%.^75^ At the same time, however, the country has practically stalled its progress in providing modern contraception to women in reproductive age. Since the year 2000, the national average –provision of modern contraception– has hardly increased from 50% to 55% in the year 2020.^76^

From the year 2000 through 2017, and according to UN estimates, Peru also managed to reduce the maternal mortality ratio, from 144 to 88 per 100,000 live births.^77^ National epidemiological surveillance records show the decline continued until 2019, but during the first year of the COVID-19 pandemic maternal mortality increased by 50%, and data up to mid-2021 suggest that by the end of the year 2021 it may increase by ∼90% compared to the 2019 level.^78^

### Future tasks

To overcome the new challenges posed by the COVID-19 pandemic, while accelerating the pace attained during two decades of progress, Peru has to prioritize maternal and reproductive health. First, by boosting the use of modern contraception methods including post-partum and long-acting reversible contraception (LARC). Second, by assuring that vaccination against COVID-19 reaches to pregnant women, or those planning to become pregnant. Fortunately, Peru has a strong national maternal mortality surveillance network that provides weekly updates to characterize the maternal mortality profile in the 24 regions in Peru. On such basis, Peru can readily design interventions according to sub-national patterns, by age or specific causes.

### Endemic communicable diseases: the case of tuberculosis and COVID-19

Communicable diseases are still endemic in Peru, exacerbating the higher demand created by the increased incidence of non-communicable diseases. The combination of non-communicable and communicable diseases occur within a socioeconomic and political context that predispose to syndemics.^79^ One emblematic case is tuberculosis. Peru is one of the countries with the most cases of tuberculosis in the Americas and it is considered as one of the high burden countries for multidrug resistant tuberculosis (MDR-tuberculosis) worldwide.^80^ The most common comorbidity for tuberculosis in Peru is diabetes.^81^ The case of tuberculosis and diabetes exemplifies the concept of syndemics where the clustering of these conditions within a population presents with persistent socio-economic inequalities.^82^ Rather than approaching each of these conditions independently, the concept of syndemics focuses on instances in which multiple health problems interact, often biologically, with each other and the sociocultural, economic, and physical environment.^82^

In the last ten years, Peru had set in place a programme to close the gaps in the tuberculosis care cascade: increased the availability of molecular diagnosis, screening of comorbidities as diabetes and HIV/AIDS, and a strengthened monitoring using an electronic registry (*Sistema de Información Gerencial de Tuberculosis-SIGTB*).^83^ During the first months of the COVID-19 pandemic in 2020, Peru detected 8,000 tuberculosis cases less than in the equivalent period in 2019.^84^ This would increase the risk of tuberculosis transmission in the community and worsen clinical presentation among those who were not timely detected. This gap in detection can be explained by the strict lockdown during March-June 2020 and patients’ fears to get infected with SARS-CoV2 if they went to health centers for tuberculosis diagnosis.

In spite of these barriers and gaps for tuberculosis control during the COVID-19 pandemic, there are potential opportunities for tuberculosis control improvement. For example, Peru had been using directly observed therapy as strategy for tuberculosis treatment since the 1990’s,^85^ but because of the lockdown, the tuberculosis program enacted different approaches to reduce treatment dropout and lost to follow-up: treatment was given for patients to take the pills at home, cell phone monitoring, home visits, among others.^86^ This gave the opportunity to explore if the use of a patient-centered approach rather than directly observed therapy would be feasible. Despite the evidence suggesting that directly observed therapy was not better than non-directly observed therapy approaches,^87^ there is resistance from local health workers to shift to other treatment delivery approaches, assuming a paternalistic approach as observed therapy is the best option to reduce the risk of lost to follow-up and MDR-tuberculosis. Nevertheless, after decades of directly observed therapy for tuberculosis care in Peru, lost to follow-up and MDR-tuberculosis remain key problems for tuberculosis control.

### Mental health

**Figure 3:**
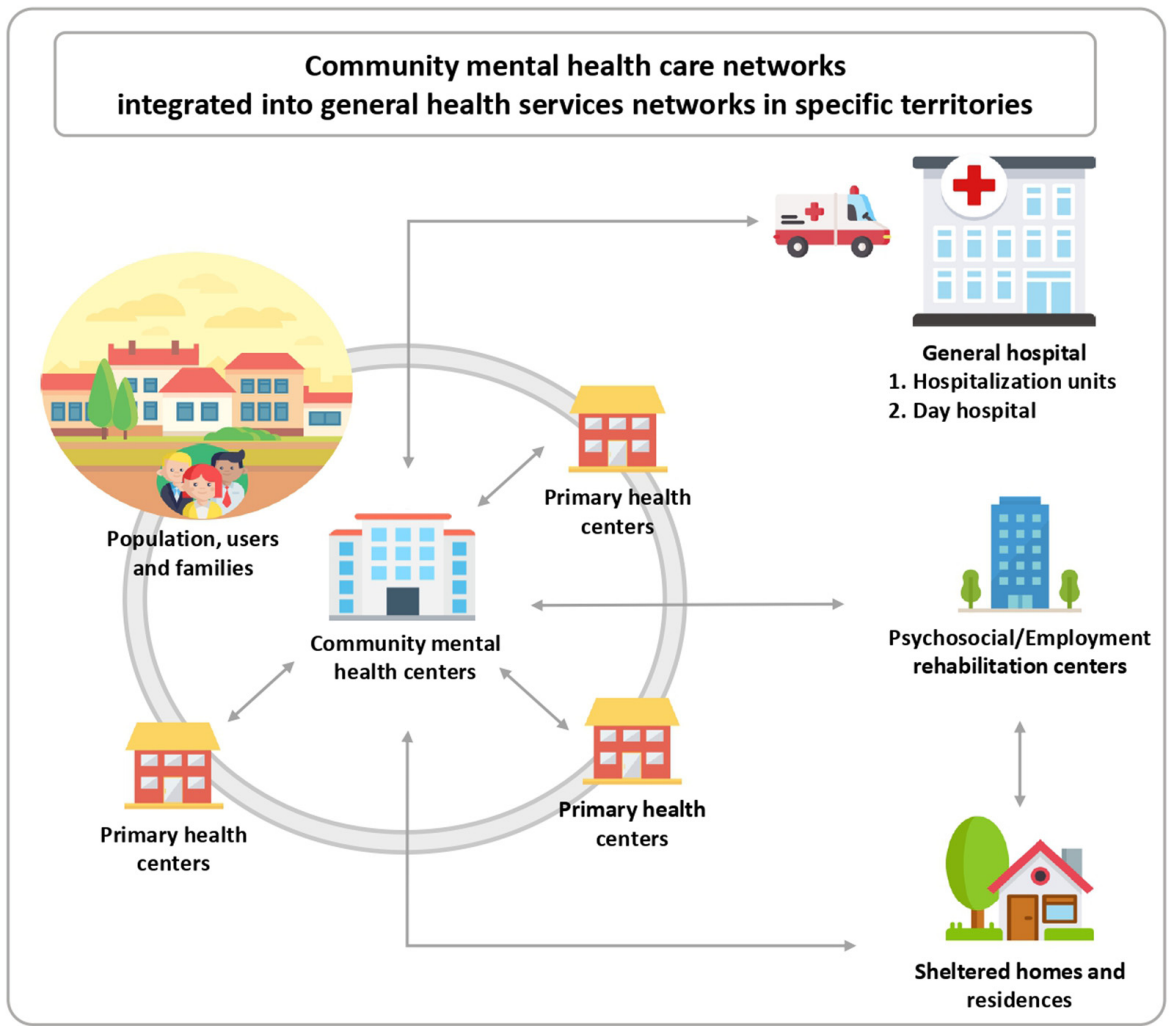
Community mental health care networks in Peru. Graphic based on icons made by Freepik from www.flaticon.com.

In Peru, mental health disorders affect one in five adults^88^ and are the main cause of burden of disease in the country.^89^ However, until a decade ago, only 10% of those who required mental health care had access to it.^88^ Since 2012, with the approval of Law 29889,^90^ Peru initiated a mental health reform that implements a community mental health care model, as part of territorial health services networks (Figure 3), aiming to reduce the existing treatment gap.^91^

### Major milestones going forward

The milestones that marked the beginning of the reform were, in 2013, the inclusion of mental health care within the public universal health insurance plan and, a year later, the creation of a results-based budget program exclusively for mental health activities. The most recent achievements of the reform are the approval of the Mental Health Law (Law 30947) and its regulations, in 2019 and 2020, respectively, which seek to guarantee the universal access to mental health care, with community, intercultural and human rights approaches.

**Figure 4:**
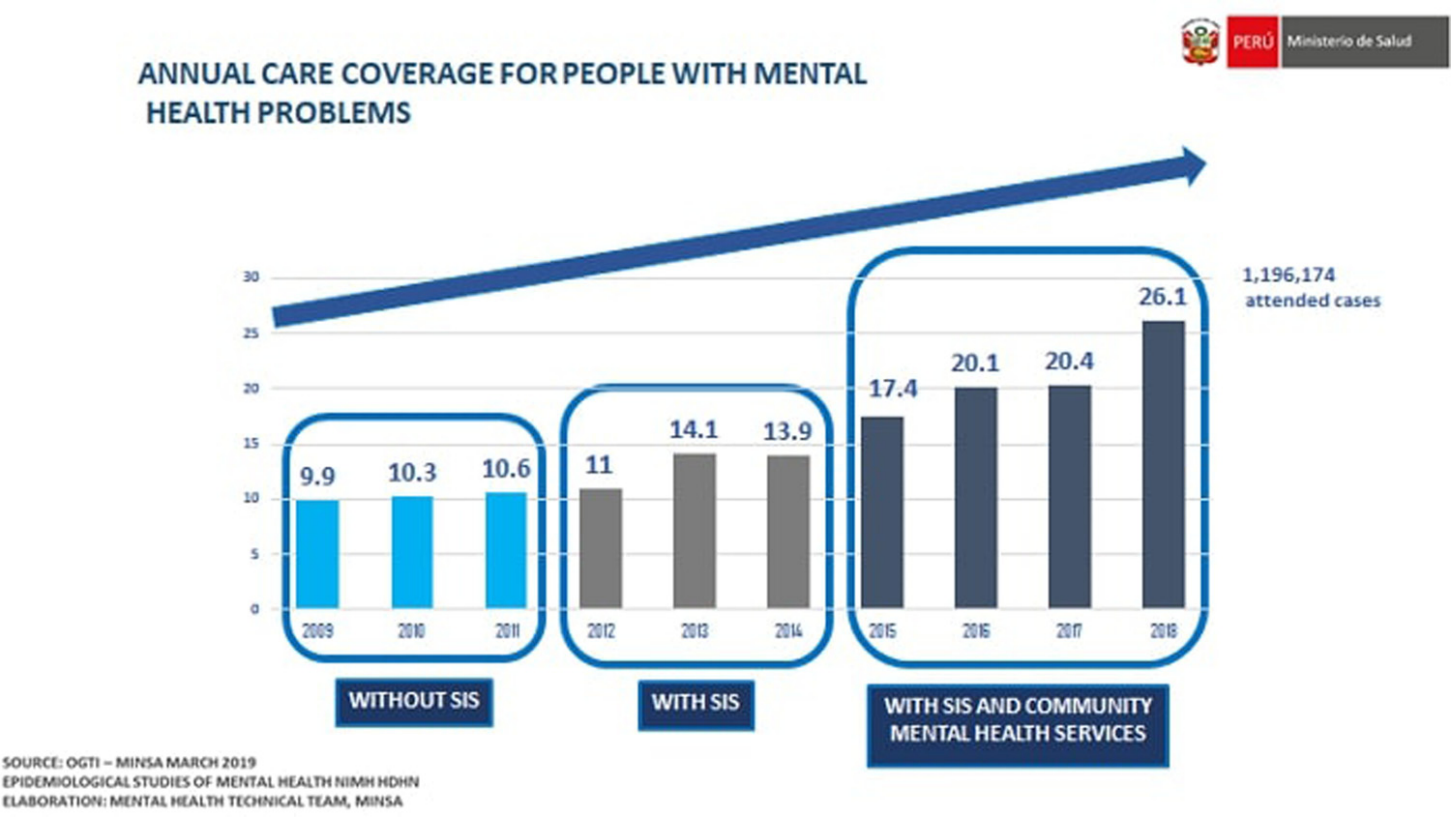
Annual care coverage for people with mental health problems, Peru 2009–2018.

During the reform, it has been possible to: (a) Develop the necessary legal and technical regulatory framework with laws, regulations, programs, plans, and technical guides, including some specific to the context of the COVID-19 pandemic;^92^^,^^93^ (b) Implement and decentralize a variety of community mental health services, through a participatory process involving different levels of the government, with more than 200 Community Mental Health Centers, 30 mental health hospitalization units in general hospitals, 48 sheltered homes for people with severe mental health disorders across the 24 regions of Peru, and psychologists in more than 1,000 health centers; (c) Progressively increase the care coverage for mental health problems (Figure 4); (d) Increase from one to 15 the list of strategic psychotropic drugs delivered free of charge; (e) Strengthen the capacities of thousands of health workers to carry out mental health promotion, prevention and recovery activities, through internships, diplomas and training in national and foreign institutions; (f) Triple the funding for mental health, from PEN 179.7 million in 2012 to PEN 558.5 million in 2021 (yet this still represents <2.5% of the health sector budget); (g) Install the National Mental Health Council, which articulates several sectors of the State to collaboratively design mental health policies, plans, and programs; (h) Establish collaborations with Latin American Ministries of Health, international organizations (e.g. PAHO), as well as with local universities, to strengthen the mental health reform with evidence, evaluate its implementation, and disseminate its progress through scientific publications.^88^^,^^94^

### Future tasks

The goal for the coming years is to consolidate the progress made so far since the introduction of the mental health reform. In addition to secure a sustained increase in financing; additional attention is required to expand and strengthen, in the short term, the offer of mental health services for more dispersed and poorer populations with the aim of reaching 281 Community Mental Health Centers, 42 mental health hospitalization units and 145 sheltered homes. Also, strengthening the articulation of these services with the primary health care, social services and community organizations, improving the training of human resources and consolidating the use of telemedicine will be needed, alongside the launch of a Mental Health Observatory.

## Non-communicable diseases

### Disease prevalence and incidence

Whilst there are solid estimates about the prevalence and incidence of hypertension^95^ and type 2 diabetes (T2DM)^96^ in Peru, we still have poor evidence about (population-based) prevalence and incidence of cardiovascular diseases (CVD) as well as cancer and chronic respiratory diseases (CRD). Regarding CVD, incidence estimates for ischemic heart disease (71 per 100,000 per year) and stroke (75 per 100,000 per year) are based on the Global Burden of Disease Study.^97^ Recently, the age-standardized population-based incidence of stroke among individuals aged ≥35 years has been estimated at 99 (95% CI 63–154) per 100,000 person-years.^98^

Information about the national burden of cancer is limited to mortality data,^99^ despite of having surveillance systems in Lima and Trujillo which inform the Globocan estimates.^100^ Finally, CRD have been mostly studied in the Andes (high altitude settings) where biomass fuels are used for cooking and home warming.^101^ Chronic obstructive pulmonary disease (COPD) prevalence has been estimated at 6%, with marked variation across settings reaching up to 10% in rural areas at high altitude.^102^

### Risk factors

Urbanization,^103^ rural-to-urban migration,^104^ and high altitude,^105^ have been studied as risk factors for non-communicable diseases in longitudinal cohorts leveraging on the unique features the Peruvian geography. In the case of T2DM, obesity may be considered the main driver with a population attributable fraction fluctuating between 33% and 75% depending on the setting and the obesity marker.^105^ In Peru, subscapular fat was among the most relevant markers to detect new T2DM cases (75%), in comparison to waist circumference (44%) and BMI (34%).^106^^,^^107^ Regarding CVD, most of the information about risk factors addressed hypertension. Obesity, either as per BMI or waist circumference, and pre-hypertension, were the main driver of hypertensive disease in Peru.^108^ In the case of CRD, urban living, high-altitude dwelling and having hypertension accounted for 26%, 21% and 16% of the overall mean annual decline in lung function, respectively.^109^

### Policies and interventions

In Peru, progress to meet the Sustainable Development Goal 3.4 has been largely informed by passive surveillance and mathematical modeling showing that Peru has improved with regards to CVD and cancer; conversely, T2DM represents a key barrier to the achievement of this goal.^110^ National policies have been focused on cancer (Plan Esperanza), allocating resources to prevention, early detection, diagnosis, treatment and care for cancer patients.^111^ Other policies focused on CRD aiming to improve access to clean fuels and “improved cook stoves”.^112^ Finally, while the implementation of the Peruvian Law on the Promotion of Healthy Eating is expected to have an impact on CVD and T2DM morbidity and mortality,^113^ such effect remains to be seen.

Local research has also shown that some interventions may be useful to tackle specific non-communicable diseases. The implementation of a salt substitute containing 25% of potassium chloride and 75% of sodium chloride reduced blood pressure levels at the population level, and halved hypertension incidence.^114^ So far, interventions for T2DM have mainly focused on secondary prevention or glycaemic control instead of prevention.^115^^,^^116^ In terms of risk factors, task-shifting interventions focusing on lifestyle behaviors delivered through mobile health have shown sustained reductions in weight after four years.^117^

### Next steps

The health system needs to move forward by establishing a more complete surveillance system for chronic non-communicable diseases, including but not limited to CVD, stroke, and cancer, but also to promote primary prevention strategies in addition to secondary prevention to reduce the burden due to complications related to non-communicable diseases. Such preventative strategies can be driven within the health sector, particularly in the primary health care setting, as well as utilising innovative approaches ‘outside’ health facilities.^114^^,^^118^ Research needs to be expanded to include implementation and evaluation of such interventions.

## Climate change and health

### Response to climate change

Peruvians currently face the impact of climate change on health due to extreme weather events such as El Niño^119^ and rising temperatures, among other hazards. The country promptly joined global climate change response efforts, creating its first National Climate Change Commission in 1993^120^ and launching the first National Climate Change Strategy in 2003.^121^ Peru hosted the 20th Conference of the Parties (2014) and adhered to the 2015 Paris Agreement, committing to pursue efforts to limit temperature increases above pre-industrial levels. In this line, Nationally Determined Contributions were issued, and then updated in 2020,^122^ aiming at reducing greenhouse gas emissions and adapting to climate change. The Nationally Determined Contributions currently aim for a 20% and 30% reduction below the 2030 business-as-usual projection of the unconditional and conditional emissions targets, respectively, although these goals are way above the 1990 and 2010 levels. Additionally, the 2018 Framework Law for Climate Change^123^ promotes clean and sustainable industries, efficient water use, territorial and environmental zoning, sustainable urban development and climate risk prevention and management. Climate Change Commissions created in 2020 in Congress^124^ and across the Executive branch,^125^ further support Peru's efforts.

### Threats of climate change

The commitment described above, however, will probably still be insufficient. Over the last three decades Peru has suffered a 167% increase in heat-related work hour losses,^126^ particularly in agriculture in coastal areas. Densely populated cities such as Lima present heat island effects, where temperatures barely above 24°C are followed by a 10–15% relative risk increase in mortality among the elderly (personal communication by co-author SMH). The incidence of climate-susceptible vector-borne diseases such as dengue will probably continue rising also, as their climate-related suitability has increased up to 6%^126^ and the presence of its vector, *Aedes aegypti,* has expanded from 48 to 89 provinces between 2005 and 2017.^127^ *A. aegypti* now thrives in cities exposed to high temperatures and heavy precipitation or droughts where adaptation plans, city risk assessments and infrastructure are lacking,^126^ leading to slower response actions and limited coordination as observed in the 2017 El Niño event.^128^ Finally, exposure to wildfires in the Amazon has increased 85% since the turn of the century and the number of days with very high/extreme fire risks rose by 24%.^126^

Greenhouse gas emission mitigation strategies are critical to improve air quality and reduce excess mortality. An estimated 6,600 premature deaths occurred countrywide in 2018 due to the excessive PM2.5 ambient air particulates; of which 1,544 were attributable to fossil-fueled land transportation and 846 to domestic sector pollution.^126^ Lima is among the most polluted cities in Latin America.^129^ Additionally, several other health-related climate change impacts must be better tracked and addressed in the future, such as tropical deglaciation, land-use change, emerging zoonotic diseases, food security and marine productivity, and access to water.

### Future work

Peru must identify climate-related risks in its cities and rural areas, and address them with evidence-based locally-adapted plans^130^ that incorporate nature-based solutions.^131^ Investing in a green and blue sustainable recovery from the COVID-19 pandemic, for example, is a unique short-term opportunity. Climate justice principles (e.g., support the right to development; share benefits and burdens equally; and decision on climate change should be participatory, transparent and accountable)^132^ should be followed to prevent climate change from magnifying inequalities in already-vulnerable Indigenous populations and climate migrants, while protecting the country's biodiversity.

## Science and technology in health

### Key achievements

Since 2005, eight programs have been publicly financed to strengthen Peru's national science and technology system. This was a consequence of a five-fold increase in Peru's investment in science, technology and innovation (STI) in the last decade, with an average of ∼US$ 280 million annually.^133^ In parallel, scientific publications in health sciences increased 8.7-fold and, particularly in the last eight years we observed a 2.5-fold increase in medicine papers and a 3-fold increase in papers about molecular biology and related fields.^8^ Through the programs Concytec/Fondecyt Researcher Incorporation, *Innóvate* and Magnet, ∼250 scientists from the best universities worldwide have been repatriated and established at academic institutions in Peru.^134^ These researchers and their local colleagues will benefit from greater opportunities that will become available through the 2019 Law for the Promotion of Scientific Researchers.^135^

### Ongoing initiatives

According to the Concytec Registry of Researchers (Renacyt), Peru has ∼5,000 researchers, which means there are 140 researchers per million people;^134^^,^^136^ this rate is smaller than in other countries from the Pacific Alliance (Chile, Colombia and Mexico) where there are ∼400 researchers per million people.^137^ Nonetheless, 31% of total Peruvian researchers work in disciplines related to the health sciences. With such strength in health research, and the incorporation of biotechnology, genetics and digital technologies, special programs are being prepared to boost research in the health sector to be funded by the Inter-American Development Bank and the World Bank (personal communication by former president of the National Council of Science, Technology and Technological Innovation). This additional investment in STI will be distributed following meritocratic criteria and based on national health priorities (personal communication by former president of the National Council of Science, Technology and Technological Innovation). This investment will also aim at improving infrastructure, increasing the availability of laboratories with higher levels of complexity and biosafety. These new resources will improve drug and medical device approval mechanisms and simplify regulations and administrative processes that hinder scientific and technological progress. Finally, a new governance system for the local scientific structure has already been approved by the government in July 2021.^138^ It aims to solve the fragmentation of the STI system, which is evident in the overlapping of funds and actions due to lack of coordination between national and sectoral actors. In addition, this new governance system might solve the persistent problem of brain drain, lack of competitive salaries, poor employment conditions for researchers, and the dearth of a clear career path for scientists in Peru. The new governance system also seeks to give continuity to STI health programs and to overcome our limitations to collect and systematize data, in order to propose strategies based on evidence and thus achieve larger societal gains in addition to the strengthening of the health system.

## Genomics, ancestry and pathogen surveillance

Over the past decade, next-generation sequencing technologies have made it possible to study Peruvian populations and infectious diseases of public health concern using novel genomics approaches that are under constant refinement.^139^

### Population studies

Reports of ancient DNA (aDNA) from archaeological sites^140^ and genomes from modern-day Native American and mestizo populations^141^^,^^142^ revealed that Peru was first populated ∼12,000 years before the present, consistent with the hypothesis that rapid early human dispersal in the Americas started some 16,000 years before the present via Beringia and moving south towards the Andes.^143^ This expansion resulted in multiple early events of admixture among communities in the Amazon, Andes, and coastal regions of Peru, all occurring well before the Incan Empire, with later evidence of European introgression following the Spanish rule that began in the 16th century^141^ The Andean Mountains have undoubtedly played a key role in selecting adaptations for high altitude. Permanent occupation of the Peruvian highlands above 2,500 meters above sea level began at approximately 9,000 before the present.^144^

### Infectious diseases

Peruvian researchers have applied next-generation sequencing technologies to (1) assess the genetic diversity of endemic and emerging pathogens in support of global genomic surveillance efforts for arboviruses,^145^, ^146^, ^147^, ^148^ antibiotic-resistant Enterobacteriaceae,^149^, ^150^, ^151^ and Mycobacterium tuberculosis;^152^^,^^153^ (2) study the genetic diversity and pathogenesis of Bartonella bacilliformis^154^^,^^155^ and B. ancashensis,^156^ the etiological agents of Carrion's Disease, a neglected bacterial infection endemic to remote high-altitude Andean valleys of Peru.^157^

### Nota bene

With the rise of SARS-CoV-2, its variants, and the threat of zoonotic viruses with pandemic potential, there is great interest in incorporating genomic and bioinformatics approaches to pathogen discovery and epidemiologic surveillance.^139^ However, to consistently generate genomic pathogen surveillance data that can inform control policies,^158^ the Peruvian genomic surveillance system urgently needs additional investments in next-generation sequencing, computing infrastructure, and improved mentoring and training of bioinformaticians at the undergraduate, graduate, and post-doctoral levels.

## Discussion

We aimed to reflect and characterize the progress made by Peru in selected health and science topics until 2021. During its independent life, Peru has experienced substantial progress including reductions in infant and maternal mortality paired with longer life expectancy. This has been achieved despite major socioeconomic crises, yet many challenges remain in place. Peru's structural deficiencies became notorious during the COVID-19 pandemic, placing Peru as one of the countries with the largest number of deaths per capita in the world.^159^

Latin America has, in general, limited investment in science and Peru lags behind other countries in the region.^160^ Despite this, researchers in Latin America and in Peru have thrived with relevant academic contributions.^161^ In this work, albeit briefly and only covering few selected topics, we aimed to position Peru in the international spotlight of science, health and research, 200 years since its independence. In so doing, we contribute to inform a better understanding of the Peruvian context and the health of Peruvians to the global health community.

This work was planned as a comprehensive overview of multiple and diverse fields related to epidemiology, public health and science in Peru. This review summarised evidence about the epidemiological transition showing the progress in reducing infectious diseases and pinpointing the growing burden of non-communicable diseases, alongside the tremendous progress to secure access to high-quality mental health care. Many of the sections in the review connect directly with primary healthcare delivery system and how this deserves much attention to improve health and wellbeing of the population. Also, throughout this article we emphasize the importance of addressing the needs of Indigenous populations. Climate change is not absent from the immediate challenges that deserve attention and concrete actions are needed. Medicine and public health, and overall Peru's development, cannot advance without the support of science. The political attention provided to public investment in science, technology and innovation in recent years appears promising towards supporting future progress in the country.

## Contributors

RMC-L and JJM conceived the idea and drafted the outline. RMC-L, WCG-V and JJM were responsible for coordinating the contributions of all authors during the drafting of the manuscript. All authors provided comments, edits and critical input. All authors approved the submitted manuscript.

## Role of the funding source

The funder had no role in study design, data collection, data analysis, data interpretation, or writing of the report.

## Data sharing

Not applicable.

## Funding

RMC-L is supported by a Wellcome Trust International Training Fellowship (214185/Z/18/Z). CZ-C is supported by the National Institute for Health Research (NIHR) and Wellcome under the NIHR-Wellcome Partnership for Global Health Research (218743/Z/19/Z). JJM acknowledges having received support from the Alliance for Health Policy and Systems Research (HQHSR1206660), Bloomberg Philanthropies (via University of North Carolina at Chapel Hill School of Public Health), FONDECYT via CIENCIACTIVA/CONCYTEC, British Council, British Embassy and the Newton-Paulet Fund (223-2018, 224-2018), DFID/MRC/Wellcome Global Health Trials (MR/M007405/1), Fogarty International Center (R21TW009982, D71TW010877, R21TW011740), Grand Challenges Canada (0335-04), International Development Research Center Canada (IDRC 106887, 108167), Inter-American Institute for Global Change Research (IAI CRN3036), National Cancer Institute (1P20CA217231), National Heart, Lung and Blood Institute (HHSN268200900033C, 5U01HL114180, 1UM1HL134590), National Institute of Mental Health (1U19MH098780), Swiss National Science Foundation (40P740-160366), UKRI BBSRC (BB/T009004/1), UKRI EPSRC (EP/V043102/1), UKRI MRC (MR/P008984/1, MR/P024408/1, MR/P02386X/1), Wellcome (074833/Z/04/Z, 093541/Z/10/Z, 103994/Z/14/Z, 107435/Z/15/Z, 205177/Z/16/Z, 214185/Z/18/Z, 218743/Z/19/Z) and the World Diabetes Foundation (WDF15-1224).

## Declarations of Interests

None. The arguments and opinions in this manuscript are solely those from the authors, and do not necessarily reflect the opinions from the institutions to which the authors belong.
